# Challenges and opportunities for outreach workers in the Prevention of Mother to Child Transmission of HIV (PMTCT) program in India

**DOI:** 10.1371/journal.pone.0203425

**Published:** 2018-09-04

**Authors:** Nishi Suryavanshi, Vidya Mave, Abhay Kadam, Savita Kanade, Srilatha Sivalenka, V. Sampath Kumar, Pauline Harvey, Radhayshyam Gupta, Asha Hegde, Nikhil Gupte, Amita Gupta, Robert C. Bollinger, Anita Shankar

**Affiliations:** 1 Lakshya, Society for Public Health Education and Research, Pune, India; 2 Johns Hopkins University, School of Medicine, Baltimore, Maryland, United States of America; 3 Division of Global HIV & TB–India Country Office, US Centers for Disease Control and Prevention, New Delhi, India; 4 National AIDS Control Organisation, New Delhi, India; 5 Johns Hopkins University, Bloomberg School of Public Health, Baltimore, Maryland, United States of America; TNO, NETHERLANDS

## Abstract

**Background:**

The Prevention of Mother-to-Child Transmission of HIV (PMTCT) program in India is one of the largest in the world. It uses outreach workers (ORWs) to facilitate patient uptake of services, however, the challenges faced by the ORWs, and their views about the effectiveness of this program are unknown.

**Methods:**

The COMmunity-Home Based INDia (COMBIND) Prevention of Mother to Child Transmission of HIV study evaluated an integrated mobile health and behavioral intervention to enhance the capacity of ORWs in India. To understand the challenges faced by ORWs, and their perceptions of opportunities for program improvement, four group discussions were conducted among 60 ORW from four districts of Maharashtra, India, as part of the baseline assessment for COMBIND. Data were qualitatively analyzed using a thematic approach.

**Results:**

Numerous personal-, social-, and structural-level challenges existed for ORW as they engaged with their patients. Personal-level challenges for ORWs included disclosure of their own HIV status and travelling costs for home visits. Personal-level challenges for patients included financial costs of travelling to ART centers, non-adherence to ART, loss of daily wages, non-affordability of infant formula, lack of awareness of the baby’s needs, financial dependence on family, four time points (6weeks, 6 months, 12 months and 18 months) for HIV tests, and need for nevirapine (NVP) prophylaxis. Social-level challenges included lack of motivation by patients and/or health care staff, social stigma, and rude behavior of health care staff and their unwillingness to provide maternity services to women in the PMTCT programme. Structural-level challenges included cultural norms around infant feeding, shortages of HIV testing kits, shortages of antiretroviral drugs and infant NVP prophylaxis, and lack of training/knowledge related to PMTCT infant feeding guidelines by hospital staff. The consensus among ORWs was that there was a critical need for tools and training to improve their capacity to effectively engage with patients, and deliver appropriate care, and for motivation through periodic feedback.

**Conclusions:**

Given the significant challenges in PMTCT programme implementation reported by ORW, novel strategies to address these challenges are urgently needed to improve patient engagement, and access to and retention in care.

## Introduction

As of 2015, India was home to an estimated 2.1 million persons living with HIV/AIDS, accounting for the 3^rd^ highest country burden of HIV in the world [1]. With India, the situation in the state of Maharashtra is of particular concern, as the state has an antenatal HIV prevalence of 0.5%, nearly twice the national average [2]. During 2014–15, 9.75 million pregnant women were provided free counselling and testing for HIV in India [3]. Furthermore, 97% of HIV-positive pregnant women and their babies received anti-retroviral (ARV) prophylaxis for the prevention to mother to child transmission (PMTCT) [3].

Despite substantial programmes, in low-middle income countries such as India, only 15% of HIV exposed infants received an HIV test within their first two months of life in 2010 [2, 4]. Furthermore, our research group at Byramjee Jeejeebhoy Medical College (BJMC) in Pune, Maharashtra, showed that only 20% of HIV-infected mothers reported exclusive breastfeeding (EBF) as their mode of infant feeding (EBF is recommended in the first six months of an infant's life as it is associated with a three- to fourfold decreased risk of HIV transmission compared to infants who were breastfed and also received other milks or foods), and only 59% of HIV-exposed infants received HIV testing by six weeks of life (unpublished hospital data). BJMC clinic counseling notes suggest this low uptake of PMTCT is due to challenges at personal, social, and structural levels. Thus, to improve the situation for individual patients, and for the national PMTCT program, it is essential to identify the contextual barriers to implementing effective interventions[5–7].

In India, PMTCT interventions started in 2002, with access to HIV testing for all pregnant women enrolled in antenatal clinics, provision of single dose Nevirapine prophylaxis for HIV positive pregnant women during labour, and for their babies immediately after birth. The HIV testing was provided through some 21,000 integrated counselling and testing centers (ICTCs) attached to antenatal clinics in public hospitals [8]. A further milestone date in India was September 2012, when the National AIDS Control Organisation (NACO), began adoption of the WHO ‘Option B’ recommendations (2010), transitioning from the single dose Nevirapine strategy to multi-drug ARV prophylaxis for pregnant mothers. Since 2013, in accordance with additional WHO guidelines (June 2013) and the recommendation of the national Technical Resource Group (December 2013) [9], NACO started implementing the more efficacious “Option B+” PMTCT program that includes four key components designed to both improve women’s health and prevent vertical HIV transmission. The components are: 1) provide lifelong ART for all pregnant and breast-feeding women living with HIV, via a triple-drug ART regimen tenofovir disoproxil fumarate plus lamivudine and efavirenz (TDF+3TC+ EFV) regardless of CD4 count or clinical stage, for their own health and to prevent vertical HIV transmission and for additional HIV prevention benefits, 2) promotion of EBF for six months; 3) administer nevirapine prophylaxis for six to twelve weeks to HIV-exposed, breastfed infants; and 4) engagement and retention of women and infants in postpartum HIV care to facilitate early infant diagnosis (EID).

The PMTCT program in India, uses outreach workers (ORW) for community mobilization to facilitate uptake of PMTCT services by HIV positive pregnant and breastfeeding women. The ORWs are recruited by local non-governmental organizations (NGOs), which are in turn identified by NACO, through State AIDS Control Societies and national partners with an effort to increase uptake of PMTCT services. The ORWs are paid an honorarium by the government through the NGOs, and receive training by NACO in the PMTCT programme, with a focus on the importance of HIV prevention, and ART initiation and adherence.

Integrated counselling and testing centers identify HIV+ women, who are then linked to ORWs. Each ICTC may have more than one ORW working in the project catchment area, depending on the HIV burden. The ORWs accompany clients to the ART center for registration, support the women with monthly home visits during pregnancy, and attend during labor and immediately afterwards, as well as up to 18 months post-delivery [10].

There is no published data available from India from the perspective of ORWs related to the barriers to PMTCT implementation services and the potential to improve programme effectiveness. This is critical to understand in order to design strategies that optimize uptake, and to reach the global goals of eliminating new paediatric HIV infections. As part of a larger research and training program, this formative research was conducted to identify and understand the primary challenges faced by ORW and translate their perspectives to strengthen training and deployment of the COMBIND research intervention.

## Methods

### Overview

We documented the challenges and opportunities facing ORWs involved in PMTCT deployment program in Maharashtra, India as part of the formative research phase in preparation for the COMmunity-Home Based INDia (COMBIND) Prevention of Mother to Child Transmission of HIV study. The trial is described below to provide context for this research. The main results of the trial to determine effectiveness of the program is forthcoming.

### Study design

The study entitled “COMmunity-Home Based INDia (COMBIND) Prevention of Mother to Child Transmission of HIV”, was a cluster randomised trial designed to determine effectiveness of the COMBIND intervention in optimizing the uptake of a PMTCT program. A cluster was the ICTC, its assigned ORWs, and their assigned pregnant and breastfeeding women.

**Fig 1:**
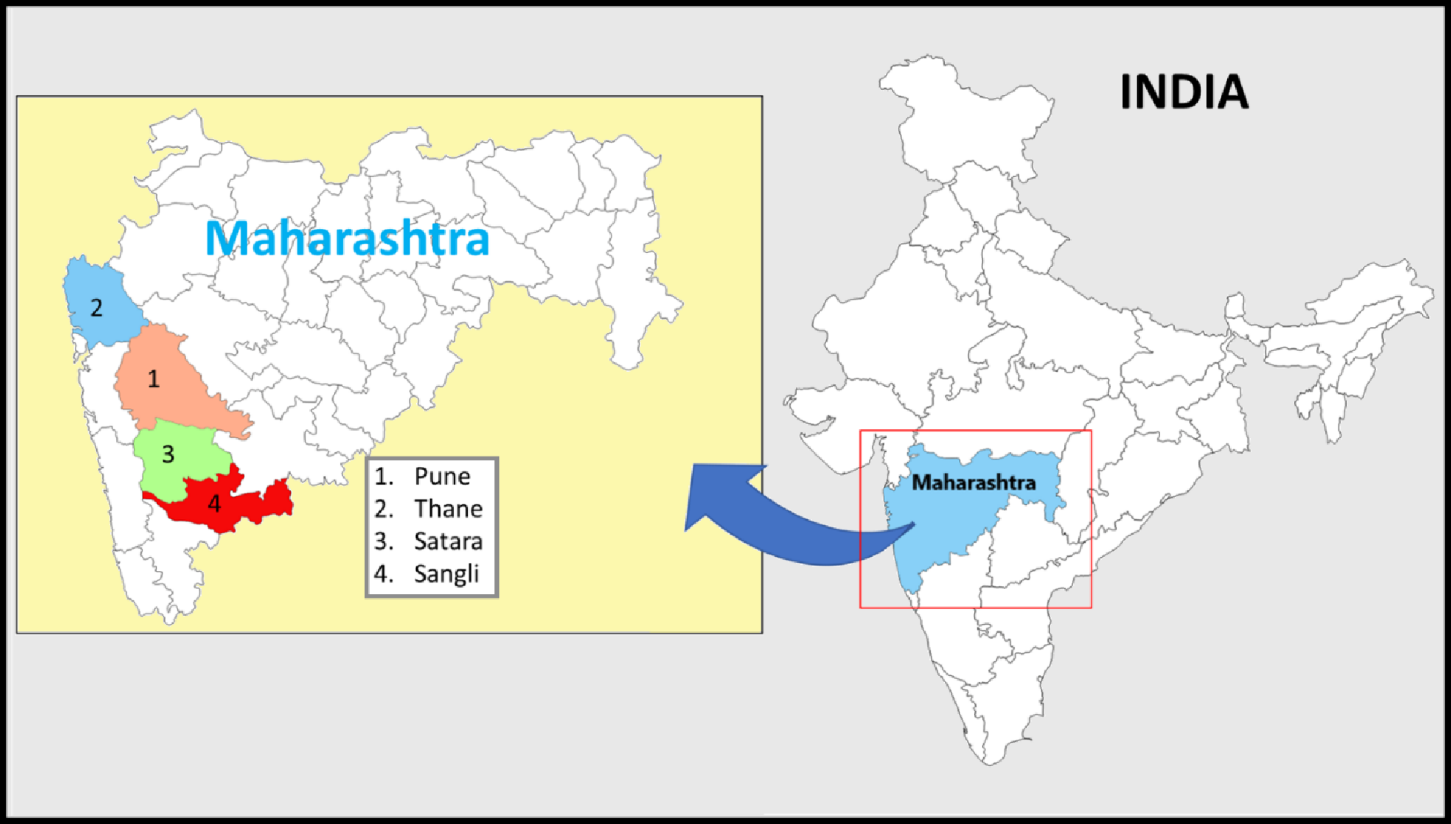
Maps of India showing the state of Maharashtra, and the four districts from which the outreach workers participated in group discussions.

**Fig 2:**
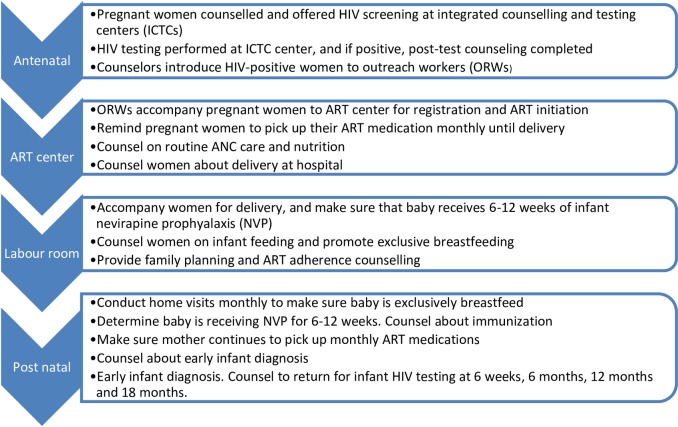
Main components of the PMTCT program, and the role of the outreach workers.

Of the 121 ORWs working with the PMTCT programme in four study districts (Fig 1) (see “study sites” below), 117 gave consent to participate in the COMBIND PMTCT study. Thereafter the 117 ORWs, (each of whom were assigned to 3–20 HIV+ mothers who were pregnant or breastfeeding), were randomized into one of two arms: (i) COMBIND intervention (n = 60) and (ii) control (providing routine care, Fig 2) (n = 57).

The COMBIND intervention was designed to facilitate consistent outreach services to pregnant/ breastfeeding women and optimize uptake of key PMTCT components by HIV positive pregnant/breast-feeding women. The integrated intervention included specialized behavioral training to improve the capacity of the ORWs, to counsel their patients, and to use mHealth technology, (an electronic mobile comprehensive health application (referred to as Emocha)). The mHealth included informative videos, in the local language, on four key PMTCT components, and counselling scripts to help HIV positive women overcome challenges they face when availing themselves of PMTCT services.

The COMBIND study was approved by Lakshya Ethics Committee and Johns Hopkins Institutional Review Board (protocol number IRB00041608).

### Study sites

The study was undertaken in the state of Maharashtra, in western and central India, because it has one of the highest prevalence’s of HIV in the country. Within the state, there is heterogeneity in rates and of the 36 districts in Maharashtra; Pune, Sangli, Satara and Thane have been chosen purposively due to high HIV prevalence and a mix of urban-rural populations.

### Discussion groups

As part of the formative stage of the COMBIND study, and to design the COMBIND intervention, it was important to understand the challenges that ORWs face while implementing the Option B+ guidelines, and what opportunities they perceived to enhance implementation of PMTCT service. Hence initial qualitative research was conducted in the four study districts (Fig 1).

Group discussions among the 60 ORW participants in the intervention arm of the COMBIND study, were conducted between July 2015 and February 2016. The group discussion sessions lasted between one and half to two hours. There was one discussion group per study district, with the number of participant ORWs per district being: Satara 11, Sangli 9, Pune 22, and Thane 18.

### Data collection

To facilitate discussion on the challenges faced by ORWs while delivering HIV care, an interview guide, (S1 File) with open-ended questions, was prepared using the framework of option B+ PMTCT cascade and responsibilities of ORWs. The focus was directed to four key components of PMTCT: (i) ART initiation and continuation, (ii) exclusive breastfeeding (EBF) until 6 months, (iii) Nevirapine (NVP) prophylaxis to baby for 6–12 weeks, and (iv) early infant HIV testing. The challenges encountered by the ORWs were classified into three categories: personal, social, and structural. The operational definition of each of these categories is presented in Table 1. To clarify and explore the challenges, we used probes such as issues related to transport accessibility for visiting ART center, non-disclosure of HIV status, stigma, fear of drug side effects, and support from family.

**Table 1:** Categorization of challenges faced by outreach workers, with levels and examples.

Category of challenge	Level	Examples
**Personal**		
	ORW at individual level	Inability to convince HIV-infected women to return to PMTCT service.
	Patient at individual level	No initiation of ART, or non-adherence to ART, due to non-disclosure of HIV status to spouse or family
**Social**		
	ORW or patients at community level	Stigma and discrimination by community members
**Structural**		
	Program or clinic level, challenges that may hinder a patient’s uptake of services	Lack of supplies, inappropriate behavior of hospital staff

After introduction of facilitators and the note taker, who are also study investigators, discussions were facilitated by three study investigators (NS, AK, and SK) who are trained anthropologists with extensive experience in conducting qualitative studies. The discussions were audio taped, transcribed in the local language (Marathi), and then translated into English. Data on socio-demographic variables of ORWs were collected by a field coordinator, using digital structured questionnaire.

### Analysis

A coding guide was prepared, which employed the three categories of challenges with respect to four key components of PMTCT: (i) ART initiation and continuation, (ii) exclusive breastfeeding (EBF) until 6 months, (iii) Nevirapine (NVP) prophylaxis to baby for 6–12 weeks, and (iv) early infant HIV testing. Data were analyzed using thematic analysis[11].

After transcription and translation, the transcripts were reviewed two to three times, and the translations were manually coded by SK and AK. The major themes of the three categories of challenge (personal, social and structural) faced by ORWs in providing the PMTCT services, had emergent sub-themes which included accessibility, stigma, facilities and supplies, and knowledge-related challenges. Themes and sub-themes were reviewed by NS, SK and AK within, and across, the four study districts, to identify cross-cutting challenges. Any disagreements between the coders were resolved through discussion. Thematic findings were illustrated using verbatim quotes which were identified as representative of a specific theme.

### Availability of data and material

The interview transcripts and their analysis are not publicly available due to ethical concerns about protecting the privacy of the participants, as the interview transcripts contained sensitive information about the participants.

## Results

Of the 60 ORWs who participated in the four group discussions, 58 were female and two were male. The median age of the ORWs was 37 years, with 53.3% having a secondary education, 91.7% had living children, and 37% reported a positive HIV status (Table 2).

**Table 2:** Sociodemographic characteristics of the 60 outreach workers who participated in the discussion groups.

Characteristic	Number	Percentage
**Age (Yrs)**		
Mean	37	
Median (IQR)	37 (32–39)	
**Religion**		
Hindu	51	85.0
Muslim	5	8.3
Buddhism	4	6.7
**Marital Status**		
Unmarried	2	3.3
Married	29	48.3
Separated / Divorced / Widowed	29	48.3
**Family type**		
Nuclear	35	58.3
Joint*	25	41.7
**Educational Status (Years)**		
Secondary (6–10)	32	53.3
Higher (11–12)	14	23.3
College (13–16)	14	23.3
**Monthly Income (Indian Rupees)**		
2,501–5,000**	36	60.0
More than 5,000	24	40.0
**Have Living Children**		
Yes	55	91.7
No	5	8.3

* A joint family in the Indian context is a family composed of a husband and wife, plus their children (married or unmarried), and grandchildren, all living together in the same house.

(1$ = 64 Indian Rupees (INR))** 2500 INR = 39$, 5000 INR = 78$

All ORWs viewed the PMTCT programme as a very important programme to prevent HIV transmission, were very satisfied with their work facilitating its implementation, and were motivated to help and make a difference in the lives of HIV-positive women.

However, all 60 of the ORWs reported experiencing significant difficulties in delivering PMTCT care. Results of our qualitative data analysis to assess the challenges faced by ORWs at three levels: personal, social and structural, presented below.

### A: Personal challenges

#### 1. Personal challenges of ORWs at individual level

ORWs faced several personal-level challenges when trying to ensure PMTCT services were appropriately used by their patients, for example ORWs sometimes disclosed their own HIV status to their patients to help patients accept their own status, understand they are not alone with HIV, and understand the importance of taking ART. The ORWs reported persistent difficulties with convincing and bringing the patients to the clinic. As one ORW stated:

When a patient comes to the antenatal clinic for the first time, she does not know anything. She is informed about routes of HIV transmission, and when she hears [about her HIV status] she is broken (par khachun geleli asate) she cries…that time we have to disclose our result to her…”see mine [result] is also positive…not to worry, you will be alright. You are not alone [HIV-positive] in this world.” This way we have to counsel her.

Most ORWs reported that patients did not accept their own HIV-positive status, had difficulties understanding anti-retroviral treatment (ART) initiation, and struggled to cope on the day ICTC counselor disclose their HIV status to them.

The ORWs felt that they did not have adequate skills to manage their relationships with their patients, to support, educate and counsel them. The ORWs from all four districts highlighted the need for improving their own training and knowledge about the rationale for infant HIV testing at four recommended time points, so that they could more effectively counsel patients to bring their babies for HIV testing. Another challenge mentioned by ORWs was lack of money for patient transport, a challenge they met by using their personal funds to bring the patients to the clinic for appointments.

One ORW from Satara was frustrated about these issues and stated:

“Many times, we spend money from our own pocket so that they come to the hospital. We accompany them. Nobody gives us that money back. Our salary is so low and most of it is spent on travelling”

The ORWs also reported that it is difficult to convince women to continue ART lifelong. Patients feel healthy, and do not see a need to continue ART life-long. One of the ORWs from Sangli narrated.

My patients ask me “Why take medicines life long? We get bored of taking medicines for 3–4 days if we have something [fever, cold or cough]…so why do we have to take it (ART) life-long?”

#### 2. Personal challenges of patients of ORW at individual level

A major patient-related personal challenge reported by ORWs in all four study districts, was the unwillingness of patients to disclose their HIV status to their spouse and family members. This lack of disclosure has significant consequences as it hindered ART adherence, and contributed to irregularity of visits at the center, and eventual loss to follow-up.

Another, treatment-related challenge faced by the patients was non-adherence to proper use of ART, due to severe medication side-effects, (dizziness, diarrhea, headache), and the complex administration instructions which require patients to take multiple tablets at specific time intervals. Patients discontinued treatment if they were not aware of what to do when side effects occurred, or if they did not understand how to take their medication. Other factors that contributed to non-adherence were time constraints, transport problems, and forgetfulness. As one ORW in Pune mentioned:

“There is a time problem, (sometimes) they just forget to take their pills. Sometimes in a hurry, they forget and leave the pill box at home, and go to the work”

Providing NVP prophylaxis to an infant was identified as a major challenge. It is challenging for the mothers to give NVP to their infants daily for six to 12 weeks in front of family members, if their own HIV status is undisclosed. Furthermore, family members often refused to give the infant NVP prophylaxis, as they were not aware of the HIV status of the parents. Three additional challenges to providing NVP prophylaxis to infants were: the extended time needed for mothers to understand why NVP prophylaxis is given to babies, patients who did not deliver their child in a hospital may refuse to initiate NVP at home, and if the mother chooses not to breastfeed, she may feel it is unnecessary to give NVP prophylaxis, due to the overall reduced risk of transmission.

Some ORWs from Sangli and Satara mentioned that access to transport was difficult, as public transport is infrequent in the rural areas of these two districts. Furthermore, patients were often reluctant to travel alone. In many cases, patient’s husbands were daily wage workers, and generally reluctant to accompany their wives to center visits, because a day’s wages would be lost during travel to and from the center.

The ORWs from all four districts reported that achieving the recommended exclusive breastfeeding was another challenge. The few HIV-positive women who decided to formula feed, were generally unable to afford it, and therefore shifted to animal milk or breast milk. If an infant’s HIV status was not disclosed to the family, it was difficult to prevent older family members (such as mother-in-law or sister-in-law) from feeding the infant sugar water, honey, or animal milk as they perceive that the mother is unable to produce sufficient breast milk. As one ORW stated:

“I visited one patient, and saw her mother-in-law feeding the three-month-old baby with cow’s milk in a bottle. I asked her why she is feeding this milk, and she told me that her daughter-in-law (patient) doesn’t get sufficient milk [breast milk] and the baby cries with hunger”

The ORWs in all districts mentioned that although mothers came for the initial infant HIV testing when the infant was six weeks old, if the initial test showed the infant was HIV negative, the mothers often refused any subsequent tests (Indian PMTCT guidelines recommends HIV testing among infants and babies at four time points, at 6 weeks as it can identify most babies who will progress rapidly and who will need life-saving ART, 6 months and 12 months depending on breastfeeding period that allows health-care providers to offer optimal care and treatment of HIV infected children, and at 18 months to make final confirmation of HIV status.).

### B: Social challenges

The ORWs stated that many patients faced social challenges that inhibited their effectiveness to link patients to care. The most widely discussed challenge in implementing the PMTCT programme was social stigma, including fear of accidental disclosure of patients’ HIV status in the community. For this reason, some patients refused to continue treatment.

The group discussions revealed a generalized community stigma toward ORWs. If an ORW visited an individual in the community who was known to be HIV-positive, the ORW might also be considered to be HIV-positive, and consequently face stigma. Several HIV negative ORWs from Satara District indicated,

“People gossip, saying that she (the ORW) comes to her house every time, and she (the ORW) is also HIV-positive. We just accept their comments and do not react”.

Due to stigma in the community, many patients did not want ORWs to visit their home and preferred to be in contact by phone. The ORWs reported that in such instances it was difficult to follow patients, as they either switched off their phone or changed the number.

Exclusive breastfeeding was not a universal practice in the study area; newborns were usually given honey or sugar water. For fear of community disclosure, women often fed their babies a mixture of breast milk and formula, not always following the recommendation of exclusive breastfeeding. One ORW from Pune stated:

“Sometimes while advising on exclusive breastfeeding, patients’ family members want us to give a guarantee that the baby will be HIV free if the baby is given only the mother’s milk. At that time, we do not know what to say”

### C: Structural challenges

The most noteworthy structural challenge was frequent drug shortages and stock-outs, especially of NVP and infant HIV testing kits. The ORWs reported that by the time supplies were once again available, patients had lost confidence in the programme, refusing to believe that medicines or testing kits were in stock. One ORW from Thane described the experiences of her patient:

“You tell me to never miss medicines, and when medicines are not available in the hospital, who is to be blamed? Why don’t you go and tell them [the hospital] [to make medicines available]”?

Other structural challenges included discriminatory staff behavior toward HIV-positive women. The ORWs reported that staff spoke rudely with them as well as with their patients. At delivery and / or post-partum, hospital staff asked the mothers how they became infected, and look at them with suspicion.

Furthermore, there were long waiting times at clinics, denial of delivery services to HIV-positive pregnant women in the rural hospitals, and lack of attention. Patients often expected that as soon as they reached the hospital, they should be seen by doctors first (before other patients), because they had small babies. Long clinic wait times were reported to demoralize women from coming for infant HIV testing. Due to these challenges, many babies were lost to follow-up before 18 months, with their HIV status and potential death from HIV remaining unknown.

The number of needle pricks required to collect sufficient blood for infant testing was also often perceived as excessive or causing illness. One ORW from Pune stated:

“In some cases, there are multiple technical pricks, the baby moves it’s hands, the baby gets fever, this [series of events] may result in problems, and it becomes difficult to convince the patient to come for their next visit”

Sometimes the mothers refused to attend follow-up visits due to lack of trained staff, and the difficulties in collecting blood from an infant (including the fact that the blood draw may cause pain). An ORW described her experience with one of her patients:

“The first time my mother-in-law accompanied me (to the clinic),and said that madam (lab technician) pricked the baby many times and collected blood, and baby cried a lot, so she (patient’s mother-in-law) told me not to go to the hospital for further testing as report is negative.”

Another major challenge to infant feeding was reported to be the conflicting advice given to women. Counsellors at the ICTC centers advised women to exclusively breastfeed, while doctors in the labour room may have advised formula feeding. The mothers were often confused and started their infant on formula after delivery. Eventually, the cost of formula feeding could become prohibitive, and only then would breastfeeding be initiated.

Regarding the perceived need to enhance the uptake of services, ORW from all districts reported that ICTC center staff should be motivated to change their behavior with patients. Equally, doctors in labour rooms needed additional training on changes in PMTCT guidelines.

The ORW stated that all these challenges pointed to the urgent need to strengthen the capacity of outreach workers to successfully implement the PMTCT programme, to sustain their motivation, and to receive additional training to enhance their skills and knowledge.

## Discussion

In India, Option B+ was rolled out in 2013, with the aim of eliminating new HIV infections among children by end of 2015 [12]. Previous studies in India [13–15], US [16, 17] and Africa [18] have reported that after HIV diagnosis, multiple social barriers cause poor initial linkage to care, low retention in care, and low rates of ART initiation and adherence. Against this background, we examined how additional challenges faced by the outreach workers in India could impede effective programme implementation. The experiences of the ORWs were key, because ORWs are critical to successful implementation of PMTCT services in India. Our study identified implementation challenges related to personal, social and structural barriers to PMTCT program service delivery, and gaps in services with respect to four key components of the PMTCT cascade.

A key personal barrier was coping with an HIV diagnosis and the need to initiate lifelong ART by the HIV positive pregnant/breast-feeding women (18). Furthermore, the women did not receive adequate counselling about the importance of ART initiation and its side effects. Non-adherence to ART was another key challenge faced by these women as reported by ORWs. The women often did not want to disclose their HIV status to their husbands and family, and did not continue medications fearing disclosure; this was a particular concern when facing the side effects of ART, when the women often felt unable to disclose their HIV status to their family, and consequently stopped taking ART. Adherence is better when HIV status is disclosed to husbands, and when both spouses are HIV positive, than those when HIV status is not disclosed to husband [19]. These findings suggest that: (i) counselling services need to be enhanced at the time of HIV diagnosis and initiation of ART, and (ii) ORWs need skills and training to effectively engage with patients to cope with diagnosis and ART. These data led to a more intense focus on strengthening counseling skills of the ORWs to more effectively communicate with their clients in this context. Specifically, we used motivational interviewing techniques [20] to provide specific strategies for ORWs to use with their patients to increase empathy, identify discrepancies in their logical framework for change and support their self-efficacy. We also designed an educational video on ART initiation and adherence as part of the COMBIND intervention.

The ORWs reported that women were commonly unable to attend clinic visits every month for ART refills due to monetary constraints, long distance to centers, and infrequent public transport, especially in the rural areas. These results were similar to those reported from Uganda[21]. Furthermore, the ORWs reported that women were reluctant to remain in care due to the rude behavior of PMTCT providers at ICTC clinics, lack of sufficient staff, lack of testing kits for babies, and drug stock-outs. Scarce human resources and delivery facilities in rural areas were significant structural barrier reported by ORWs from the rural districts in our study. Similar problems have previously been reported elsewhere in India [22] and in Africa [23], and clearly pose a major challenge for ORWs to facilitate the service uptake of these women. These data helped us to develop standardized counseling scripts to address these specific challenges and would be available to the ORWs through their tablet computers. The use of mobile alerts to remind HIV positive pregnant/breast-feeding women of their upcoming appointments was also seen as an important strategy to encourage them to maintain their clinic schedules. Hence it is important to develop interventions to help both the ORWs and the women they serve, by providing training and information to find alternate ways to handle and overcome these structural challenges.

Giving NVP prophylaxis to infants was problematic from the mother’s perspective. The ORWs reported that the mothers raised concerns about potential side effects of the drug, had a perception that their baby was healthy and therefore did not need NVP, and had concerns about disclosure of HIV status to other family members. Similar findings were reported in a study from Tanzania [24]. Furthermore, the ORW noted that convincing HIV-infected women to exclusively breastfeed was difficult. Two main factors contributed to this. Firstly, exclusive breastfeeding is not a universal social norm in India. Commonly infants in India are given honey, sugar water, and gutti (a semi-solid paste of almonds) at birth and continue receiving these throughout infancy. Secondly, clinicians attending a delivery often advised women to formula feed. While clinicians may focus on the risks of HIV transmission through breastfeeding, counsellors and ORW focus on the beneficial effect of exclusive breastfeeding, also advising formula feeding is not in alignment of current WHO PMTCT guidelines. This resulted in conflicting advice being given to women. Findings from Uganda [25] were similar, highlighting the importance of appropriate and on-going training of healthcare staff, so that new mothers are well-informed and able to make their own decision. Using standardized counseling scripts with the correct medical advice on NVP prophylaxis and exclusive breastfeeding was seen as essential to promote appropriate care. This reinforced the need to include a dedicated mobile information support system for the ORWs that included videos on importance of NVP prophylaxis and exclusive breastfeeding and counselling scripts in local language.

The final issue identified in this formative research was related to HIV testing of infants. The ORWs reported that mothers were demotivated from bringing their babies for HIV testing due to lack of knowledge about baby’s HIV testing at four time points, lack of availability of testing kits, and lack of personnel trained to collect blood from babies for HIV testing. These results are consistent with those reported from South Africa [26] and Uganda [21]. Loss to follow up of HIV exposed infants is reported to be 29%, according to the Indian PMTCT program [27] A study in Nigeria [28] suggested the utility of cross-cutting interventions, including recruitment and retention of motivated healthcare workers in PMTCT implementation and training. Utilizing these data, we developed fourth educational video on importance of EID testing and retention in post-partum care.

The on-going frustrations described by the ORWs in this formative research were evident. Therefore, the behavioral training intervention for COMBIND was developed to support the ORW’s self-efficacy and personal empowerment as well as the empowerment of the HIV+ pregnant and postpartum women. This included a specialized behavioral training focused on strengthening personal agency using psychological techniques from psycho-analysis, cognitive-behavioral therapy, and mindfulness[29] and information-motivation-behavioral skills model of care initiation and maintenance (sIMB-CIM) [30]. This aspect of the ORW training was designed to increase mental clarity for problem solving, reduce emotional stresses and increase motivation.

The range and magnitude of challenges reported by the ORW prompted the design of innovative interventions using a mobile health platform (mHealth). The material included role plays, counselling scripts and videos on how to help patients to navigate barriers. For example, if a patient report non-adherence to ART/ NVP prophylaxis, or missing EID visits due to lack of transport, the ORWs are prompted to motivate patients to come up with their own solution, by asking them what they would do if they faced an emergency, how transport could be arranged in such cases?

The finalized PMTCT Intervention included the following components: These included: 1) Specialized behavioral training for the ORWs; 2) Pretested PMTCT counseling/education scripts and videos; 3) A tablet-based mobile health application designed to support the collection of data from the HIV positive pregnant/breast-feeding women during home visits, facilitate use of the scripts and videos, optimize communication between ORWs and patients, and provide ORWs and mothers access to appointment alerts.

The behavioral education for the ORWs in the COMBIND arm included an intensive one-week residential training course designed to assist ORWs engagement with HIV+ women. Education was comprised of personal empowerment exercises designed to increase ORWs awareness of themselves and increase their motivation and confidence for the work. In addition, ORWs engaged in active learning techniques, practice counseling strategies including motivational interviewing. Field Coordinators met monthly in person with ORWs to provide feedback on the rate of missing visits and examine strategies to support the mother’s improved health seeking.

Special counseling scripts and videos were developed in the local language, designed to improve the four prioritized program outcomes and to provide information about the 4 key PMTCT components. The scripts helped the ORWs provide consistent messaging during their counseling of HIV+ mothers and assisted them in overcoming barriers to optimize PMTCT care.

ORWs in the COMBIND arm were provided tablet computers and training on how to operate the tablets and the study application. The COMBIND mHealth application was designed to support delivery of the COMBIND intervention and assist communication between ORWs and patients to optimize uptake of PMTC services. All content on the application was in the local language (Marathi). The mHealth application was also designed to send automatic SMS alerts to cell phones of the ORWs and enrolled mothers.

The main limitation of our study is that the results may not be generalizable to a larger population, or to other Indian states due to cultural differences. Furthermore, many of the structural challenges that the ORWs reported, such as non-availability of HIV testing kits for babies, and drug stock-out that were beyond the reach of the consequent COMBIND intervention.

Despite these limitations, this study of the challenges reported by the ORWs helped in developing a training module and intervention addressing information gaps and minimizing the challenges of implementing PMTCT services. The goal of such training was to help the ORWs better assist HIV positive mothers to navigate the challenges they faced in taking advantage of PMTCT services. This mobile health intervention was supported by behavioral training that focused on enhancing personal agency and motivation of ORW and providing them with communication skills to more effectively engage patients.

## Supporting information

S1 File Focus group discussion guide. (PDF)
