# Supplementary material for: Challenges and opportunities for outreach workers in the Prevention of Mother to Child Transmission of HIV (PMTCT) program in India
Source: PLoS One. 2018 Sep 4;13(9):e0203425. doi: 10.1371/journal.pone.0203425 (PMC6122806; doi:10.1371/journal.pone.0203425)
Supplement: S1 File — (PDF) [file pone.0203425.s001.pdf]

Group discussion guide: ORWs

After greeting the group and introduction of note takers and facilitator start discussion using following guide.

- Tell us about the HIV testing services provided by the Integrated Counselling and Testing Centers (ICTCs) to pregnant women in your area? (who counsel, pre and posttest counselling, acceptance of testing, acceptance of positive results, patient's reaction)
- How does HIV/AIDS affect pregnant/breastfeeding women in your community?
- In your opinion, what challenges you faced in the transition from current PMTCT program to Option B+?
- What do you think about Option B+ service delivery guidelines and practices that help or hinder (probe for physical, financial, challenges) access to PMTCT services.
- Tell us any barriers HIV positive pregnant and breastfeeding women face to access PMTCT services (Probes: Since HIV diagnosis to initiate ART, to bring baby for HIV testing, infant feeding, problems at clinics, accessing care, distance travelled, family support etc).
- How do you help them?
- What are challenges to exclusive breastfeeding? (Probe: what is meaning of EBF, how cultural context influence decision of EBF? How disclosure of HIV status influence EBF)
- What are the difficulties/challenges that you are encountering in rolling out Option B+? (linking pregnant women to ART clinic, starting ART, NVP for babies, bringing them for follow up visits, HIV status disclosure, issues in community like stigma and discrimination).
- How beliefs, knowledge and practices of patients affect their uptake of ART, infant HIV testing, exclusive breastfeeding? (probe further for any misconception and how it hinders your work?)
- How you overcome these challenges?
- In your opinion, what is expected from ORWs toward pregnant/breastfeeding women, including referral services related to NVP prophylaxis, CD4 Count, and Early infant diagnosis?
- How ICTC centres and ART centres are rolling out this program? Do you face any issues when you link women and their babies to these services?
- How confident are you (communication/counselling skills, training provided by program) in negotiating uptake of PMTCT services by women? (Probe for reasons).

- Do you have any need for further training/skills?
- What else do you think is needed to overcome these challenges and improve PMTCT uptake?

Major Themes and subthemes:

Structural Challenges

- Lack of support at ART/ICTC clinic
- Lack of supplies
- Lack of medicines
- Behaviour of staff
- Lack of staff
- Lack of PMTCT knowledge among staff

Personal challenges

- Non- disclosure of HIV status
- Lack of money
- Transport
- acceptance of HIV status
- Knowledge and training gaps
- Fear of side effects

Social Challenges

- Stigma
- Lack of support
